# Developing a Comprehensive Scale for Parenting Resilience and Adaptation (CPRA) and an assessment algorithm: a descriptive cross-sectional study

**DOI:** 10.1186/s40359-022-00738-3

**Published:** 2022-02-22

**Authors:** Shoko Sugao, Kei Hirai, Masayuki Endo

**Affiliations:** 1 Clinical Psychology, Graduate School of Human Sciences, Osaka University, Yamadaoka, 1-2 Suita, Osaka, 565-0871 Japan; 2 Graduate School of Medicine, Osaka University, Yamadaoka, 1-7 Suita, Osaka, 565-0871 Japan

**Keywords:** Comprehensive Scale for Parenting Resilience and Adaptation, Mothers, Development, Cognitive and behavioural characteristics, Child-rearing, Parenting, Depression, Japan

## Abstract

**Background:**

Adapting to child-rearing is affected by multiple factors, including environmental and individual factors. Previous studies have reported the effect of a single factor on childcare maladjustment; however, to prevent maladaptation in and to support child-rearing, a comprehensive evaluation of factors is necessary. Therefore, this study developed a comprehensive assessment tool for childcare adaptation.

**Methods:**

We conducted semi-structured interviews with specialists whose jobs entailed supporting parents. Items were extracted from the interview data and used to develop a new questionnaire. Mothers with a child aged 0–3 years completed the Quick Inventory of Depressive Symptomatology as a depression index. We performed both factor and correlation analyses on the collected, data and multiple regression analyses to determine which factors predict depressive tendencies leading to childcare maladaptation. Subsequently, an assessment algorithm model was built.

**Results:**

1,031 mothers responded to the questionnaire which had 118 items in five domains. A factor analysis was performed on each domain to develop the Comprehensive Scale for Parenting Resilience and Adaptation (CPRA). The CPRA comprised 21 factors and 81 items in five subcategories: Child’s Temperament and Health (1 factor, 5 items); Environmental Resources (5 factors, 20 items), Perceived Support (4 factors, 15 items); Mother’s Cognitive and Behavioural Characteristics (6 factors, 22 items), and Psychological Adaptation to Parenting (5 factors, 19 items). Correlations between all factors and depressive symptoms were identified. Depressive symptoms were predicted by factors from four subcategories: Environmental Resources, Perceived Support, Mother’s Cognitive and Behavioural Characteristics, and Psychological Adaptation to Parenting. A comprehensive model of mothers’ psychological adjustment was developed using the CPRA’s domain structure.

**Conclusions:**

The CPRA enables researchers to understand the strengths and weaknesses of mothers. Mother’s maladaptive states can potentially be predicted by understanding the interactions between these multiple factors. The developed model can provide the necessary support to mothers and increase mothers’—and others’—awareness of the support that can prevent childcare maladjustment.

## Background

Mothers’ mental and physical health has a significant impact on child-rearing behaviour and affects the development of their children. Pregnancy and childbirth can be both physical and psychological stressors for mothers [1, 2]. Therefore, it is necessary to establish appropriate support for mothers, particularly mothers who struggle with child-rearing; various efforts are being made to provide support [3–5]. Predicting maladaptation to child-rearing, preventing maladaptation, and preparing the necessary resources that can be shared during prenatal check-ups can help ensure the appropriate support for mothers. Most often, support and intervention for mothers have been provided based on the experience of clinicians, such as doctors, midwives, nurses, public health nurses, and clinical psychologists. By observing the condition of both the mother and the child and communicating with the mother, these clinicians assess the traits and development of the mother and child and may discern struggles or challenging situations. When support is deemed necessary, intervention and support measures—customised according to the needs of each mother caring for her baby—can be offered. The Edinburgh Postnatal Depression Self-Evaluation Scale (EPDS) has been used to identify mothers who need early support [6]. However, this scale addresses only depression, and limited information is available that can be used to develop interventions for anxiety and other difficulties. Other limitations to the EPDS have also been highlighted [7, 8].

Previous studies of maladjustment to child-rearing have focused on individual factors, such as family support [9–11], mothers’ fatigue and characteristics, disabilities [12, 13], and child development. However, few studies have comprehensively and quantitatively examined environmental factors and factors related to the child, rather than focusing on maternal development and personality traits as predictors of early child-rearing maladjustment. Assessing mothers’ personality/developmental factors and environmental factors around childbirth can promote maternal self-care and prepare support coordination by supporters. Baraitser and Noacl [14] defined maternal resilience as the capacity for mothers to survive the vicissitudes of the parenting experience itself and stated it has received little attention. An existing measure of childcare resilience in Japan is the Childcare-Related Resilience Scale by Miyano et al. [15]. As this scale consists of three factors, "I am", "I have" and "I can", which were identified as components of resilience by Grotberg [16] and Heiw [17], it does not include the developmental characteristics of mothers and the nature of the children they are raising. Therefore, this study developed the Comprehensive Scale for Parenting Resilience and Adaptation (CPRA). CPRA can be utilised to comprehensively assess maternal developmental/personality traits and environmental factors related to parenting maladjustment. To develop the scale, predictive factors for childcare maladjustment were extracted from previous research reviews and interviews with clinicians. Using the extracted items, a quantitative survey was conducted involving mothers raising children. We present a structured model of developmental/personal traits and environmental factors that predict mothers’ maladaptation to childcare.

## Methods

### Participants

Participants were mothers raising a child aged 0–3 years old and who live in Japan. The sample size was set at 1200, based on the guidelines for the number of items included in the multivariate analyses—118 items × 10 [18]. The survey was distributed to 4800 people who met the sample conditions set by the research company INTAGE Inc., and 1031 valid responses from participants who provided informed consent were included in the final analyses (response rate of 21.5%). Sampling was performed so that the questionnaire would be distributed in urban and rural areas of Japan.

*Survey period*: October 2019.

### Procedure

The survey was conducted as a web survey. An original questionnaire was developed, and a cross-sectional study was conducted to assess the psychological adjustment of each mother. The questionnaire comprised 138 items, including 4 face items. Of these, 118 questions were original to the study’s questionnaire, and 16 items were from the Quick Inventory of Depressive Symptomatology (QIDS). The completion time was approximately 30 min. On the face sheet, we asked about socio-demographic characteristics, such as age, history of pregnancy and childbirth, child’s age, employment, family stressors, and household structure. Next, mothers were asked to answer 118 questions that were created based on the results of interviews with specialists in child-rearing support to measure the factors related to the difficulty of raising children. Each item was rated on a scale ranging from 1 (*strongly disagree*) to 5 (*strongly agree*).

In creating questionnaire items for inclusion, we first examined factors from previous studies that had been found to affect feelings associated with difficulties raising children. It became clear that the primary factor categories were environment, children, and personality. Next, an interview survey was conducted with four experienced specialists (one liaison nurse, one clinical psychologist, and two social workers) involved in childcare support at obstetrics and gynaecology and childcare support centres. In the interview, each specialist was asked to explain in detail the characteristics of cases encountered in the clinical setting that demonstrated a need for support. Interview data were used in exploratory content analysis. From this analysis, as many question items as possible, that could broadly cover the study’s research focus were created. Three researchers examined the appropriateness of these potential questions, grouping them according to the primary factor categories identified from previous research. Items were reduced to a number that could be answered reasonably, and the wording and ease of answering were confirmed by researchers. A questionnaire was created that consisted of 118 items in 5 domains: Child’s Temperament and Health, Environmental Resources, Perceived Support, Mother’s Cognitive and Behavioural Characteristics, and Psychological Adaptation to Parenting. In order to establish appropriate support for mothers struggling with childcare, the Japanese version of the QIDS (QIDS-J) [19] was used as an index for the mental maladjustment of the mothers, rather than resilience or adjustment scales. The QIDS is commonly used as a clinical outcome measure. Although it would have been best to examine the concurrent validity of the CPRA with the EPDS, due to a large number of items in the CPRA itself, the predictive validity of the CPRA was validated by the use of the QIDS.

### Statistical analysis

The demographic characteristics and backgrounds of the participants were summarized using descriptive statistics. Independent exploratory factor analyses were performed using the maximum likelihood method and the Promax rotation method for the five domains of comprehensive psychosocial variables. After extracting the factor structure from several candidates and the interpretability in each domain, item analyses were performed considering factor-loading scores and correlation coefficients between items within the same factor to thereby select the proper items by measuring their factors and excluding redundant items. We performed a factor analysis of the remaining items after item selection to confirm the final factor structure. In addition, we calculated the Cronbach’s α and the factor score for each factor belonging to each domain.

Finally, to confirm content and predictive validity, we performed a hierarchical linear regression on the final items to predict QIDS-J scores as an index of mothers’ psychological adaptation and distress. All statistical analyses were conducted using IBM SPSS Statistics 26.

### Ethical approval

This study has been approved by the Ethics Review Committee of the Graduate School of Human Sciences, Osaka University (No.19010), and the Ethics Review Committee of Observation Research, Osaka University Hospital (No.19290–2).

## Results

### Participant backgrounds

The survey included 1,031 participants with a mean age of 31.7(± 5.3) years and a mean number of children was 1.17 (Table 1).

**Table 1:** Demographic characteristics of the participants (*N* = 1031)

N = 1031	Mean	*SD*	Range
Number of conceptions	1.27	0.5	[1–6]
Number of spontaneous miscarriages	0.24	0.57	[0–6]
Number of artificial abortions	0.15	0.46	[0–4]
Number of stillbirths	0.01	0.13	[0–3]
Age of child (months)	19.23	12.8	[0–47]
Number of children living together	1.17	0.38	[1–3]

	*n*	%
Fultime job	315	30.6
Parttime job	153	14.8
Caregiver to elderly	3	0.03
Chronic condition	49	4.9
Husband's illness	26	2.5
Special care of older children	232	22.5
Primipara	757	73.4
Perinatal loss	306	29.7
Has two or more children	170	16.5

### Factor structure and items of comprehensive psychosocial variables

The exploratory factor analyses and item analyses for 118 items in five domains yielded a one-factor structure (5 items) for the category Child’s Temperament and Health, a five-factor structure (20 items) for Environmental Resources, a four-factor structure (15 items) for Perceived Support, a six-factor structure (22 items) for Mother’s Cognitive and Behavioural Characteristics, and a five-factor structure (19 items) for Psychological Adaptation to Parenting (Table 2).

**Table 2:** Results of factor analyses for CPRA items

Child’s Temperament and Health	Child’s Temperament and Health
	I
My child often seems uncomfortable when I hold him/her	0.679
My child is generally in a good mood*	− 0.616
My child cries often	0.550
My child gets sick easily	0.438
My child does not eat much (including feeding and baby food)	0.357

Environmental Resources	Relationship with the Medical Staff	Partner Temperament	Parental Autonomy	Partner Autonomy	Child-Rearing/Long-Term Care Burden
	I	II	III	IV	V
I think the healthcare workers are reliable*	**0.933**	0.055	− 0.015	0.013	0.001
I can trust the healthcare workers*	**0.906**	0.014	0.012	− 0.026	0.008
I can communicate well with the healthcare workers at the regular health checkup for my child*	**0.550**	0.057	0.063	0.084	0.007
I can't decide on the family doctor for my child	**− 0.355**	0.062	0.053	0.059	0.085
My husband (partner) is a difficult person	0.020	**0.893**	0.008	0.092	− 0.014
My husband (partner) is very particular about something	0.058	**0.702**	− 0.002	0.155	− 0.006
I am often at the mercy of my husband (partner)	− 0.008	**0.536**	0.003	− 0.203	0.024
My husband (partner) can control his emotions well*	0.011	**− 0.454**	0.004	0.280	0.033
My parents are good at time management*	− 0.067	0.027	**0.882**	− 0.018	0.021
My parents are good at keeping things tidy and in order*	− 0.094	0.047	**0.726**	− 0.008	0.053
My parents can take care of themselves*	0.175	− 0.021	**0.483**	− 0.017	− 0.057
My parents are healthy*	0.112	− 0.095	**0.434**	0.011	− 0.050
My husband (partner) is good at time management*	0.015	0.060	− 0.023	**0.717**	− 0.004
My husband (partner) is good at keeping things tidy and in order*	− 0.046	0.145	− 0.021	**0.702**	0.080
My husband(partner) can take care of himself*	0.013	− 0.135	− 0.021	**0.682**	− 0.018
My husband (partner) is healthy*	0.084	− 0.182	0.114	**0.325**	− 0.054
(If you have an older child) the older child needs much care	0.077	− 0.039	− 0.021	− 0.039	**0.867**
(If you have an older child)The older child is regressing	0.047	− 0.062	0.006	− 0.047	**0.780**
(If you have an older child)The older child has an illness or disability	− 0.140	0.068	0.004	0.103	**0.434**
I care for my parents or grandparents	− 0.167	0.060	0.029	0.103	**0.328**

Perceived Support	Husband's/Partner's Support	Parental Support	Lack of Psychological Support from Husband/Partner	Sufficient Social Support
	I	II	III	IV
My husband (partner) takes care of our child*	**0.933**	0.005	0.145	− 0.047
My husband (partner) does housework*	**0.807**	0.011	0.150	− 0.097
My husband(partner) is uncooperative in raising children, and I feel lonely	**− 0.681**	− 0.002	0.227	− 0.006
I feel loved by my husband(partner)*	**0.664**	− 0.053	− 0.064	0.120
My parents help me with childcare*	0.002	**1.023**	− 0.028	− 0.155
I can rely on my parents for childcare and housework*	− 0.023	**0.921**	− 0.079	− 0.082
My parents often gives me advice about childcare*	0.047	**0.571**	0.064	0.290
I have someon outside my family who can help me raise my child*	− 0.054	**0.210**	− 0.129	− 0.044
My husband (partner) takes care of our child, but I sometimes feel lonely	0.148	− 0.148	**0.714**	− 0.023
My husband (partner) does not understand me as a mother	− 0.297	− 0.005	**0.657**	− 0.065
I have a good relationship with my parents (both families)*	0.004	0.260	0.067	**0.691**
My parents and I have different ways of thinking and methods of raising children, which makes me stressful	0.095	0.162	0.121	**− 0.523**
I have a friend who can talk about parenting*	0.020	0.164	− 0.065	**0.230**
Relationships with mom friends(including online) are stressful	0.030	0.101	0.163	**− 0.221**
I can use the information on the internet about child-rearing*	0.096	0.020	0.070	**0.213**

Mother’s Cognitive and Behavioural Characteristics	Inattentiveness	Emotional Control	Systemization Urge	Simultaneous/Overall Processing	Social Intolerence	Attachment Problems
	I	II	III	IV	V	VI
I often forget something	**0.754**	− 0.051	0.002	0.076	− 0.081	0.037
I often make careless mistakes	**0.714**	0.054	0.099	0.002	− 0.160	− 0.019
I often miss hearing	**0.616**	− 0.054	0.036	− 0.112	− 0.045	− 0.050
Although I received explanetions over and over, I don't understand well	**0.435**	0.020	0.024	− 0.154	0.197	− 0.107
I am not bothered or upset when my child doesn't do what I want*	0.117	− **0.685**	0.010	0.182	0.138	0.039
When I get frustrated, I can calm myself down*	− 0.034	− **0.660**	− 0.014	0.075	0.185	0.008
It is unavoidable that things do not go as planned*	0.083	− **0.600**	− 0.074	0.094	− 0.003	0.063
I often feel so negative that I don't want to see my child's face	− 0.036	**0.433**	− 0.135	− 0.017	0.186	0.091
I have my ideal form of child-rearing plan, and I want to apply it somehow	− 0.021	0.013	**0.832**	− 0.063	− 0.053	0.003
I have an idea that things should be in its right place, and I try to apply it	0.056	0.009	**0.774**	0.054	− 0.120	0.006
I get confused when things don't go as planned	0.067	0.109	**0.478**	− 0.126	0.025	− 0.011
I want to complete one thing until I am satisfied	− 0.003	− 0.155	**0.453**	− 0.066	− 0.011	− 0.017
I'm good at doing more than one thing at a time*	− 0.028	− 0.026	− 0.121	**0.767**	0.085	0.013
I am good at chatting while doing errands*	0.102	− 0.032	− 0.129	**0.716**	− 0.012	− 0.026
With just a little talk, I know what the other person is trying to do*	− 0.109	− 0.091	0.148	**0.433**	0.038	− 0.050
It is difficult for me to understand a person's facial expression	0.135	0.085	− 0.070	− **0.304**	0.070	0.020
I don't understand the explanations of the healthcare wokers such as doctors during the regular health checkup for my child	0.063	0.019	− 0.141	− 0.029	**0.628**	− 0.089
It is painful for me to wait in a noisy place such as a medical examination waiting room	− 0.157	0.141	− 0.080	0.056	**0.566**	0.022
I can't calm down unless someone is there	− 0.024	0.157	− 0.032	0.017	**0.501**	− 0.015
Even if I receive advice, I want to raise my child in my own way as much as possible	− 0.066	− 0.115	0.089	0.126	**0.375**	0.013
When I was a child, my parents (or major caregivers) didn't take care of me	− 0.010	− 0.008	− 0.002	− 0.069	− 0.065	**0.965**
When I was a child, I was treated violently by my parents(or major caregivers)	− 0.019	0.088	− 0.056	0.031	0.023	**0.590**

Psychological Adaptation to Parenting	Lack of Self-Confidence	Possibility of Coping	Love for the Child	Self-Esteem	Self-Responsibility
	I	II	III	IV	V
I have a lot of concerns about parenting	**0.882**	0.042	− 0.073	0.001	− 0.001
Sometimes I don't know what to do about parenting	**0.880**	0.008	− 0.013	− 0.027	− 0.027
I often get lost when raising children, and I'm worried whether it's correct	**0.868**	0.043	0.059	0.060	− 0.010
I'm not confident in myself	**0.318**	− 0.172	0.170	− 0.029	0.095
I feel that I have time to spend freely*	0.039	**0.824**	− 0.025	− 0.127	− 0.009
I feel that I get relaxed for any length of time*	0.072	**0.758**	0.093	− 0.032	− 0.034
I release my stress moderately*	− 0.096	**0.745**	0.011	0.016	0.051
I think I manage my time well*	− 0.019	**0.571**	− 0.060	0.206	0.076
I love my children*	− 0.006	− 0.013	**0.955**	− 0.002	0.033
I strongly want to protect my children*	0.023	− 0.034	**0.855**	0.003	0.023
Having children makes me feel warm*	0.024	0.067	**0.692**	0.030	− 0.018
I can overcome difficulties*	− 0.063	− 0.042	0.006	**0.804**	0.051
I am able to do what is good for my child's health*	− 0.001	− 0.005	0.004	**0.629**	0.032
I can understand my feelings by myself*	0.048	0.158	− 0.012	**0.597**	− 0.044
I am proud of myself raising a child*	0.073	− 0.094	0.037	**0.525**	− 0.107
I think it's my fault that my child doesn't stop crying	− 0.003	0.065	0.076	− 0.028	**0.889**
I feel like my child's cry is blaming me	0.021	0.023	− 0.019	− 0.008	**0.868**
I feel empty when I'm raising a child	0.203	− 0.113	− 0.213	− 0.017	**0.388**
I feel like I'm not valued	0.165	− 0.178	0.044	0.041	**0.246**

Reverse scored items are indicated by with *

The numbers that indicate a higher loading on each factor are in bold

### Child’s Temperament and Health

The exploratory factor analysis and the item analysis of seven items in the category Child’s Temperament and Health identified a one-factor structure with five items, with appropriate interpretability (e.g. *My child often seems uncomfortable when I hold him/her*; Cronbach’s α = 0.645).

### Environmental Resources

The exploratory factor analysis and the item analysis of 33 items in the Environmental Resources domain yielded a four-factor structure with 20 items with appropriate interpretability. The subscales were interpreted as follows: (1) Relationship with the Medical Staff (e.g. *I think the healthcare workers are reliable*; Cronbach’s α = 0.757), (2) Partner Temperament (e.g. *My husband [partner] is a difficult person*; Cronbach’s α = 0.740), (3) Parental Autonomy (e.g. *My parents are good at time management*; Cronbach’s α = 0.735), (4) Partner Autonomy (e.g. *My husband [partner] is good at time management*; Cronbach’s α = 0.706), (5) Child-Rearing/Long-Term Care Burden (e.g. *[If you have an older child], the older child needs much care*; Cronbach’s α = 0.701).

### Perceived Support

The exploratory factor analysis and the item analysis of 15 items in the Perceived Support domain yielded a four-factor structure with 15 items with appropriate interpretability. The subscales were interpreted as: (1) Husband’s/Partner’s Support (e.g. *My husband [partner] takes care of our child*; Cronbach’s α = 0.846), (2) Parental Support (e.g. *My parents help me with childcare*; Cronbach’s α = 0.753), (3) Lack of Psychological Support from Husband/Partner(e.g. *My husband [partner] takes care of our child, but I sometimes feel lonely*; Cronbach’s α = 0.671), (4) Sufficient Social Support (e.g. *I have good relationships with my parents*; Cronbach’s α = 0.496).

### Mother’s Cognitive and Behavioural Characteristics

The exploratory factor analysis and the item analysis of 41 items in the Mother’s Cognitive and Behavioural Characteristics domain yielded a six-factor structure with 22 items with appropriate interpretability. The subscales were interpreted as (1) Inattentiveness (e.g. *I often forget something*; Cronbach’s α = 0.741), (2) Emotional control (e.g. *I am not bothered or upset when my child does not do what I want*; Cronbach’s α = 0.649), (3) Systemisation Urge (e.g. *I have my ideal form of child-rearing plan, and I want to apply it somehow*; Cronbach’s α = 0.691), (4) Simultaneous/Overall Processing (e.g. *I am good at doing more than one thing at a time*; Cronbach’s α = 0.673), (5) Social Intolerance (e.g. *I do not understand the explanations of healthcare workers, such as doctors, during the regular health check-up for my child*; Cronbach’s α = 0.577), (6) Attachment Problems (e.g. *When I was a child, my parents [or major caregivers] did not take care of me*; Cronbach’s α = 0.731).

### Psychological Adaptation to Parenting

The exploratory factor analysis and the item analysis of 22 items in the Psychological Adaptation to Parenting domain yielded a four-factor structure with 19 items with appropriate interpretability. The subscales were interpreted as: (1) Lack of Self-Confidence (e.g. *I have a lot of concerns about parenting*; Cronbach’s α = 0.824), (2) Possibility of Coping (e.g. *I feel that I have the time to spend freely*; Cronbach’s α = 0.813), (3) Love for the Child (e.g. *I love my children*; Cronbach’s α = 0.863), (4) Self-Esteem (e.g. *I can overcome difficulties*; Cronbach’s α = 0.731), (5) Self-Responsibility (e.g. *I think it’s my fault that my child doesn’t stop crying*; Cronbach’s α = 0.783).

### Relationships between psychological maladjustment on the QIDS and the CPRA domains

#### Correlations with QIDS

The Pearson’s product-moment correlations between the 21 factors on the CPRA’s five domains (i.e. Child’s Temperament and Health, Environmental Resources, Perceived Support, Mother’s Cognitive and Behavioural Characteristics, and Psychological Adaptation to Parenting) and QIDS were calculated. As shown in Table 3, significant correlations with the QIDS were observed for all factors, including weak ones (*p* < 0.01). In the domain of Mother’s Cognitive and Behavioural Characteristics, the factors of Social Intolerance (*r* = 0.344), Emotion Control (*r* = 0.318), Inattentiveness (*r* = 0.264), and Attachment Problems (*r* = 0.256) were significantly correlated with the QIDS. Weak correlations were also found between the factors Simultaneous/Overall Processing (*r* = 0.182) and Systemisation Urge (*r* = 0.128) and the QIDS.

**Table 3:** Correlations between CPRA and QIDS

	Inattentiveness	Emotional Control	Systemization Urge	Simultaneous/Overall Processing	Social Intolerence	Attachment Problems	Husband's/Partner's Support	Parental Support	Lack of Psychological Support from Husband/Partner	Sufficient Social Support	Relationship with Medical Staff
Inattentiveness	1										
Emotional control	0.195	1									
Systemization urge	.174**	.210**	1								
Simultaneous/overall processing	.343**	.216**	− 0.06	1							
Social intolerence	.282**	.299**	.136**	.178**	1						
Attachment problems	.101**	.208**	0.023	.131**	.247**	1					
Husband's/partner's support	− .095**	− .191**	− 0.02	− .078*	− .101**	− .144**	1				
Parental support	− 0.01	− .199**	− .064*	− .130**	− .092**	− .291**	.095**	1			
Lack of psychological support from husband/partner	− .177**	− .276**	− .146**	− .120**	− .211**	− .207**	.485**	.159**	1		
Sufficient social support	− .102**	− .344**	− 0.01	− .294**	− .300**	− .394**	.199**	.442**	.235**	1	
Relationship with medical staff	.065*	.271**	− 0.06	.156**	.252**	.183**	− .195**	− .153**	− .171**	− .381**	1
Partner temperament	.193**	.093**	0.016	.133**	.131**	.173**	− .513**	− 0.01	− .390**	− .161**	.135**
Parental autonomy	0.052	.175**	− 0.01	.089**	0.06	.234**	− .119**	− .244**	− .112**	− .288**	.203**
Partner autonomy	0.016	.159**	0.043	0.047	.095**	.119**	− .517**	− .105**	− .248**	− .184**	.158**
Child-rearing/long-term care burden	0.049	.159**	− .077*	.099**	.103**	.176**	− .109**	− 0.01	− .122**	− .214**	.147**
Child’s temperament and health	.186**	.301**	− 0	.242**	.203**	.142**	− .152**	− .128**	− .245**	− .298**	.264**
Lack of self-confidence	.269**	.374**	.199**	.171**	.294**	.102**	− .199**	− .117**	− .433**	− .207**	.149**
Possibility of coping	.100**	.309**	0.039	.172**	.096**	.135**	− .286**	− .253**	− .374**	− .305**	.165**
Love for the child	0.024	.338**	− .168**	.222**	.189**	.247**	− .123**	− .163**	− .104**	− .362**	.302**
Self-esteem	.170**	.398**	− 0.04	.351**	.173**	.164**	− .137**	− .153**	− .161**	− .367**	.305**
Self-responsibility	.160**	.478**	.135**	.161**	.377**	.257**	− .280**	− .160**	− .496**	− .368**	.247**
QIDS score	.264**	.318**	.128**	.182**	.344**	.256**	− .239**	− .139**	− .402**	− .298**	.207**

	Partner Temperament	Parental Autonomy	Partner Autonomy	Child-Rearing/Long-Term Care Burden	Child’s Temperament and Health	Lack of Self-Confidence	Possibility of Coping	Love for the Child	Self-Esteem	Self-Responsibility
Inattentiveness										
Emotional control										
Systemization urge										
Simultaneous/overall processing										
Social intolerence										
Attachment problems										
Husband's/partner's support										
Parental support										
Lack of psychological support from husband/partner										
Sufficient Social support										
Relationship with medical staff										
Partner temperament	1									
Parental autonomy	.090**	1								
Partner autonomy	.287**	.131**	1							
Child-rearing/long-term care burden	.102**	.120**	0.046	1						
Child’s Temperament and Health	.183**	.156**	.115**	.285**	1					
Lack of self-confidence	.177**	.071*	.094**	.068*	.329**	1				
Possibility of coping	.194**	.191**	.258**	.133**	.266**	.343**	1			
Love for the child	.081**	.217**	.077*	.300**	.305**	.067*	.178**	1		
Self-esteem	.085**	.170**	.151**	.207**	.341**	.225**	.393**	.352**	1	
Self-responsibility	.258**	.122**	.149**	.185**	.415**	.611**	.385**	.282**	.325**	1
QIDS score	.262**	.103**	.146**	.187**	.256**	.439**	.352**	.200**	.254**	.521**

* means p < 0.05 and ** means p < 0.01

In the domain of Perceived Support, the QIDS was strongly correlated with Psychological Support from Husband/Partner (*r* = −0.402). It was also significantly correlated with Sufficient Social Support (*r* = −0.298) and Husband’s/Partner’s Support (*r* = −0.239). In addition, a weak correlation was found between the QIDS and the Parental Support (*r* = −0.139) factor.

In the domain of Psychological Adaptation to Parenting, there was a strong correlation between the QIDS and Self-Responsibility (*r* = 0.521) and Lack of Self-Confidence (*r* = 0.439). Significant correlations were also found between Possibility of Coping (*r* = 0.352), Self-Esteem (*r* = 0.254), and Love for the Child (*r* = 0.200) and the QIDS scores.

In the domain of Environmental Resources, a significant correlation was found between the QIDS and the factors of Partner Temperament (*r* = 0.262) and Relationship with Medical Staff (*r* = 0.207). A weak correlation was found between the QIDs and the factors Child-Rearing/Long-Term Care Burden (*r* = 0.187), Partner Autonomy (*r* = 0.146), and Parent Autonomy (*r* = 0.103). It was also significantly correlated with the Condition of a Child (*r* = 0.256) factor in the domain of Child’s Temperament and Health.

#### CPRA factors predicting QIDS scores in the hierarchical multiple regression analyses

Table 4 shows the results of the six-step hierarchical multiple regression analyses that included the demographic variables and the factor variables from the CPREA to predict QIDS scores as the index for participants’ psychological maladjustment. Model 1 comprised only demographic variables, Child’s Temperament, and Health was entered in Model 2, the Environmental Resources variables in Model 3, the Perceived Support variables in Model 4, Mother’s Cognitive and Behavioural Characteristics variables in Model 5, and Psychological Adaptation to Parenting in Model 6.

**Table 4:** Hierarchical regression results for QIDS scores

Variables	Model 1		Model 2		Model 3		Model 4		Model 5		Model 6	
	β	*p*	β	*p*	β	*p*	β	*p*	β	*p*	β	*p*
Age	− .106	.001	− .123	< .001	− .100	.001	− .107	< .001	− .056	.051	− .047	.087
First birth	− .041	.384	.010	.817	.110	.020	.086	.055	.086	.049	.057	.167
Miscarriage/stillbirth experience	.041	.193	.052	.089	.036	.225	.017	.547	.008	.769	.021	.406
Having two more children	− .032	.465	− .020	.637	− .012	.767	− .020	.601	− .029	.445	− .036	.312
Age of youngest children	.014	.676	− .001	.967	− .045	.166	− .050	.107	− .079	.009	− .065	.024
Child’s Temperament and Health			.267	< .001	.163	< .001	.096	.002	.049	.103	− .039	.184
*Environmental Resources*												
Relationship with Medical Staff					.105	.001	.044	.145	.014	.631	.017	.541
Partner Temperament					.194	< .001	.106	.001	.089	.005	.076	.012
Parental Autonomy					.028	.342	− .012	.673	− .019	.501	− .022	.418
Partner Autonomy					.042	.168	− .003	.924	− .006	.852	− .005	.865
Child-Rearing/Long-Term Care Burden					.152	< .001	.119	< .001	.116	.001	.101	.002
*Perceived Support*												
Husband's/Partner's Support							.013	.735	− .005	.885	.006	.855
Parental Support							− .033	.302	− .018	.550	− .006	.839
Lack of Psychological Support from Husband							− .283	< .001	− .220	< .001	− .084	.011
Sufficient Social Support							− .153	< .001	− .077	.027	− .031	.362
*Mother’s Cognitive and Behavioural Characteristics*												
Inattentiveness									.098	.001	.100	< .001
Emotional Control									.120	< .001	.004	.890
Systemization Urge									.036	.201	.030	.267
Simultaneous/Overall Processing									.010	.727	.005	.870
Social Intolerence									.146	< .001	.103	< .001
Attachment Problems									.066	.027	.069	.015
*Psychological Adaptation to Parenting*												
Lack of Self-Confidence											.122	< .001
Possibility of Coping											.132	< .001
Love for the Child											.030	.324
Self-Esteem											.015	.636
Self-Responsibility											.232	< .001
*R*^2^	.013	.004	.082	< .001	.161	< .001	.253	< .001	.316	< .001	.393	< .001
Adj *R*^2^	.008		.077		.152		.253		.302		.377	
Δ*R*^2^	.013	.022	.070	< .001	.080	< .001	.095	< .001	.063	< .001	.078	< .001

In the final model, the amount of change in *R*^2^ was significant, then the age of the youngest child, Partner Temperament, Child-Rearing/Long-Term Care Burden, Lack of Psychological Support from Husband/Partner, Inattentiveness, Social Intolerance, Attachment Problems, Lack of Self-Confidence, Possibility of Coping, and Self-Responsibility were significant predictors of the QIDS score (β = -0.065, *p* = 0.024; β = 0.076, *p* = 0.012; β = 0.101, *p* = 0.002; β = − 0.084, *p* = 0.011; β = 0.100, *p* < 0.001; β = 0.103, *p* < 0.001; β = 0.069, *p* = 0.015; β = 0.122, *p* < 0.001; β = 0.132, *p* < 0.001; β = 0.232, *p* < 0.001, respectively).

## Discussion

To our knowledge, this is the first study to create a comprehensive assessment tool to investigate the psychosocial characteristics of perinatal mothers. The most important finding is that we identified five domains of characteristics: Child’s Temperament and Health, Environmental Resources, Perceived Support, Mother’s Cognitive and Behavioural characteristics, and Psychological Adaptation to Parenting. Two domains—Environmental Resources and Perceived Support—have been identified in previous studies [20]. However, Mother’s Cognitive and Behavioural Characteristics has not been used for comprehensive evaluations in previous studies; therefore, this is an original finding of this study. Assessing these domains may lead to specific and tangible support for mothers after childbirth from various health care professionals. For example, the cognitive and behavioural characteristics measured by CPRA may be related to existing individual characteristics that typically lead to difficulties and stress in situations other than parenting. Understanding mothers’ vulnerabilities at the outset of parenting could help predict long-term adaptation and enable healthcare professionals to develop countermeasures. The domains of Child’s Temperament and Health, Environmental Resources, and Perceived Support contribute to better understanding the kind of supportive environment the mother experiences.

**Fig. 1:**
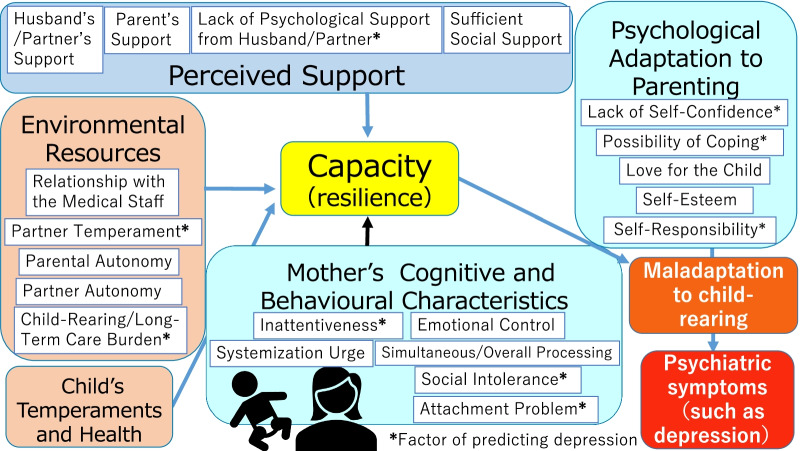
Comprehensive model of psychological adaptation based on CPRA

The second important finding is that among these domains, Partner Temperament, Child-Rearing/Long-Term Care Burden, Lack of Psychological Support from Husband/Partner, Inattentiveness, Social Intolerance, Attachment Problems, Lack of Self-Confidence, Possibility of Coping, and Self-Responsibility are factors that significantly explained participants’ depressive tendencies on the QIDS. A study using the Edinburgh Postnatal Depression Scale, another measure of depression that includes questions on emotions, such as fun, happiness, anxiety, and fear, demonstrated an association between depression and factors such as child temperament, husband support, and self-esteem [21–23], consistent with the results of this study. Therefore, the CPRA appears to be a valid evaluation tool for predicting mothers’ mental health issues, including depression (Fig. 1). A notable strength of the CPRA is that it can comprehensively measure both a mother’s emotional status and detailed cognitive and behavioural aspects of her mental health, such as Inattentiveness, Social Intolerance, Attachment Problems, and the presence of sufficient social support.

The results of the CPRA can be utilised in clinical practice. For example, healthcare practitioners can implement measures such as informing the mother, who are at risk for depression, of the specific factor of concern at the time of the examination. Moreover, for mothers who are influenced by external factors, such as psychological support from their partners, it is possible to propose psychological education for both the mother and family. Furthermore, other health professionals working with mothers can use this tool to assess mothers’ cognitive and behavioural characteristics, as these may be difficult for general healthcare professionals to evaluate. This requires the development of an educational programme for evaluation, in addition to the CPRA.

The study is limited by its cross-sectional design. Longitudinal studies are necessary for the future to determine whether the domains and factors measured by this scale predict maternal maladaptation. Our team is currently working on a project to create a cohort of patients who have experienced their childbirth in one perinatal unit to evaluate the prognosis longitudinally.

## Conclusions

Maladaptation to parenting is caused by a combination of multiple factors. Understanding how these factors interact via the CPRA makes it possible to predict the probability. When a mother is predicted to fall into child-rearing maladjustment, a comprehensive model can be used to show concretely and specifically what kind of support is needed for that mother. The mother, her family, and her child-rearing supporters can implement measures to prevent the mother from becoming maladjusted. Similarly, it is possible to understand which support is most effective in the event of childcare maladjustment. As this study focuses on the child-rearing behaviour of mothers, researching the child-rearing behaviour of fathers may lead to results that further support parents’ challenges and struggles related to child-rearing difficulties. In addition, the influence of culture on the results needs to be considered in the future. Especially during a pandemic where antenatal and postnatal services are likely to be affected.

## Data Availability

The datasets during and/or analysed during the current study available from the corresponding author on reasonable request.

## Abbreviations

CPRA: Comprehensive scale for parenting resilience and adaptation
EPDS: Edinburgh postnatal depression self-evaluation scale
QIDS: Quick inventory of depressive symptomatology
